# Thyroid Ultrasonography: Much Ado About Nothing? A Provocative Analysis

**DOI:** 10.3390/cancers17111764

**Published:** 2025-05-24

**Authors:** Petra Petranović Ovčariček, Luca Giovanella

**Affiliations:** 1 Department of Oncology and Nuclear Medicine, University Hospital Center Sestre Milosrdnice, 10010 Zagreb, Croatia; p.petranovic@gmail.com; 2 School of Medicine, University of Zagreb, 10010 Zagreb, Croatia; 3 Department of Nuclear Medicine and Thyroid Center, Gruppo Ospedaliero Moncucco, Clinica Moncucco, Via Soldino 10, 6900 Lugano, Switzerland; 4 Clinic for Nuclear Medicine, University Hospital of Zürich, 8091 Zürich, Switzerland

**Keywords:** thyroid ultrasonography, overdiagnosis, thyroid cancer, clinical guidelines, healthcare resources, thyroid nodules, thyroid incidentalomas, thyroid dysfunction, diagnostic imaging, cost-effectiveness

## Abstract

Thyroid ultrasonography (US) usage has risen significantly over the past two decades, with annual increases of up to 21% in some healthcare systems. This review examines patterns in thyroid US usage, factors driving potential misuse, and strategies to mitigate overuse. While thyroid US provides valuable information on thyroid morphology and structure without radiation exposure, inappropriate use—estimated at 10–50% of exams—leads to adverse consequences, including patient anxiety, unnecessary procedures, and potential overdiagnosis of thyroid cancer. The widespread adoption of US has coincided with increased thyroid cancer diagnoses, yet mortality rates remain unchanged, suggesting overdiagnosis rather than actual disease increase. Clinical guidelines consistently recommend selective US use not for routine evaluation of thyroid dysfunction (hyper/hypothyroidism) without palpable abnormalities, but for the anatomical assessment of palpable nodules. For thyroid incidentalomas (ITNs), evidence suggests negligible malignancy risk for nodules < 1 cm, arguing against further investigation. The paper proposes a rational approach to thyroid US, emphasizing that patients with thyroid dysfunction without palpable abnormalities, euthyroid patients without palpable nodules, and patients with subcentimetric ITNs should not undergo thyroid US. Addressing this overutilization requires a better understanding of contributing factors and targeted interventions. By restricting US to appropriate clinical scenarios, healthcare resources can be optimized without compromising patient outcomes, ensuring that rare cases of clinically significant thyroid cancer receive proper diagnosis and treatment.

## Simple Summary

This paper examines the concerning trend of excessive thyroid ultrasound usage in healthcare today. Thyroid ultrasounds have become increasingly common, often performed without clear medical necessity, leading to the identification of harmless nodules that trigger unnecessary anxiety, additional testing, and even overdiagnosis of thyroid cancer. Our paper reviews when thyroid ultrasounds are truly needed versus when they are excessive, pointing out that these scans offer little benefit for most patients without clinically evident nodularity. We aimed to provide practical guidelines for ordering and performing thyroid ultrasounds. By reducing unnecessary scans, healthcare resources can be better directed, and patients can avoid needless worry and procedures while ensuring those with genuine thyroid concerns receive appropriate care. This paper suggests how thyroid conditions should be managed, prioritizing clinical judgment over routine imaging.

## 1. Introduction

The use of thyroid US has substantially risen over the past two decades, reflecting a global trend. A study combining data from the Medicare and Surveillance, Epidemiology, and End Results (SEER) databases revealed an annual increase of 21% in US use over a decade (2002–2013) [1]. Similarly, an analysis within the Veterans Affairs healthcare system demonstrated a nearly five-fold increase in the rate of US use, from 125/100,000 persons in 2001 to 572/100,000 in 2012. Particularly, the widespread adoption of the US has reshaped the approach to managing thyroid conditions. Notably, the incidence of thyroid cancer has risen dramatically in recent decades, mostly as a result of an increase in papillary thyroid carcinoma.

The causes of the rise in thyroid cancer incidence are controversial: some authors worry about the role of unknown environmental risk factors, while many authors are concerned about overdiagnosis. A recent study compared incidence and mortality in China and G20 countries. Between 1990 and 2019, thyroid cancer incidence increased by 289.6% in China, with a more minor increase in the G20 group, especially among women over 50. Patients < 70 years old showed the worst prognosis, but unfortunately, histotypes were not evaluated. Interestingly, they noted a significant relationship between thyroid cancer incidence and prognosis with obesity. Additionally, iodine deficiency is known to increase the number of more aggressive follicular thyroid cancers (compared to papillary ones). Overall, they recognized that the increasing trend may be largely attributed to overdiagnosis, but suggested the inclusion and evaluation of environmental and genetic factors, in order to identify patients requiring more extensive evaluation [2,3]. On the other hand, many studies demonstrated the predominant increase in small (indolent) papillary thyroid cancers after the introduction of thyroid US in clinical practice [4,5]. Udelsman and colleagues analyzed data from the United States Cancer Statistics 1999–2009 to evaluate different incidences across the United States and to assess the cause of differences. Even if thyroid cancer incidence increased in every state, the pattern was not uniform. Notably, the highest incidences were found in locations with the highest density of endocrinologist and US procedures. Moreover, the frequency of cervical US and thyroid cancer incidence rates were significantly correlated for both males (r = 0.40, *p* = 0.0091) and females (r = 0.36, *p* = 0.0197) [6]. Studies from Korea also indicate that the increase in thyroid cancer diagnoses can be attributed to extensive screening and overdiagnosis [7]. Ahn and colleagues conducted a comprehensive analysis examining how thyroid cancer screening in South Korea related to thyroid cancer detection rates, cancer subtypes, and mortality outcomes. Their findings revealed remarkably high thyroid cancer incidence rates during 2008–2010, with 64.1 cases per 100,000 people overall—showing a pronounced gender disparity (21.1 in males versus 107.3 in females). Crucially, they demonstrated a clear geographical correlation: regions with higher screening rates consistently showed higher cancer incidence rates, establishing a direct relationship between screening intensity and cancer detection without corresponding mortality benefits. Moreover, thyroid screening was uniquely associated with detection of papillary thyroid cancer and, remarkably, was not associated with mortality due to thyroid cancer. The authors concluded that “*the extent to which opportunistic thyroid cancer screening is converting thousands of asymptomatic persons to cancer patients without any known benefit to them needs to be examined carefully*” [8]. Interestingly, the level of thyroidectomy-related regret in patients with clinically low-risk papillary thyroid microcarcinoma and the determinants of decision regret were evaluated by Li G and colleagues [9]. They showed that 319 (24.2%) of those who undergo thyroidectomy and 4 (3.4%) who undergo active surveillance (AS) express heightened regret based on validated decision regret scores in the first online survey (*p* < 0.001). Multivariable analysis and the second online survey for patients with heightened regret confirm that postoperative lower thyroid cancer-specific quality of life (QoL) (scar and psychology) (75.5%) is the most common factor for heightened regret of thyroidectomy, followed by preoperative understanding of disease (15.0%), presentation of complications (3.8%) and other factors (5.7%). Overall, these results highlight that more caution should be exercised when low-risk micro-PTC patients are scheduled for thyroidectomy and further corroborate that no additional diagnostic actions are necessary in such patients. Variable data were reported from different European countries. Van den Bruel and colleagues conducted a retrospective population-based cohort study to investigate whether regional variation in cancer incidence was associated with different thyroid disease management. Again, they showed that the rate of imaging was higher in the high incidence regions [10]. Vaccarella and colleagues compared the expected and observed cases of thyroid carcinoma in different countries and found a significant excess of detected cases in South Korea, Italy, United States and France and a moderate excess of observed cases in UK and Scotland, while the expected and observed cases overlapped in Nordic Countries, Australia and Japan, respectively [5].

All in all, also accounting for regional differences (i.e., iodine intake, genetic and environmental variables), a clear increase in differentiated thyroid cancer diagnosis is demonstrated worldwide and linearly associated with the availability and diffusion of thyroid US. Admittedly, available epidemiological data are not yet available for the last decade, and a trend toward an initial reduction was observed in the United States and South Korea, respectively [11,12,13]. All in all, such data suggest that changing clinical practices are likely to have led to the recent decline in thyroid cancer diagnoses. A similar reversal was observed with prostate cancer incidence in the 1990s, after the adoption and subsequent decrease in widespread prostate-specific antigen screening [14]. These observations support the role of US in determining the overdiagnosis of thyroid carcinoma and the importance of rationalizing the use of the method in clinical practice. It should certainly not be forgotten that US plays a crucial role in evaluating clinically relevant thyroid nodules and, above all, in the pre-operative evaluation and follow-up of patients with thyroid cancer [15]. However, inappropriate use of this technology can induce adverse consequences for patients and unjustified increase in costs and should be avoided.

Edwards and colleagues carried out a systematic review and meta-analysis of seven selected studies (1573 individuals). The overall frequency of inappropriate thyroid US use was 46%. The pooled frequency of inappropriate US in patients with thyroid dysfunction (either hypothyroidism or thyrotoxicosis) was 17% and the frequency due to nonspecific symptoms without a palpable mass was 11%. Notably, no study examined interventions to address inappropriate use [16]. Liel and colleagues examined how primary care physicians use thyroid US before referring patients to an endocrine clinic and reviewed 69 patients that underwent US at their endocrine clinic. Documented reasons for US were suspected thyroid mass (*n* = 35, 51%), thyroid dysfunction (*n* = 21, 30%), neck pain (*n* = 5, 7%), dyspnea (*n* = 4, 6%), and dysphagia (*n* = 2, 3%). Overall, of the 69 US cases reviewed, 64 (93%) were not appropriate [17]. Hueber and colleagues evaluated data from primary care ambulatory care (2012–2017) in Bavaria, Germany, with 13 million inhabitants. Patients who underwent TSH received early US within 28 days from the TSH test (observation group) or TSH test, but no early US (control group). Propensity score matching was used and socio-demographic characteristics, morbidity and symptom diagnosis were adjusted (*n* = 41,065 per group after matching). The authors’ analysis revealed four distinct patterns of thyroid testing behavior. The first group, comprising nearly 25% of patients, underwent minimal testing with approximately two TSH tests and no US. A second group (16.6%) received more intensive blood testing with about five TSH tests but still no imaging. The majority of patients (55%) fell into a third category characterized by moderate testing—roughly three TSH tests combined with two thyroid US. A small minority (6.2%) experienced remarkably intensive evaluation with nearly 11 TSH tests and 4 US tests. A critical pattern emerged regarding timing: in the higher-utilization groups, US typically occurred early in the diagnostic process. Specifically, over 80% of patients in the third group and 75% in the fourth group underwent thyroid US at the beginning of their evaluation. This early US use—often without clear clinical indication—was associated with a troubling cascade effect: these patients subsequently underwent more thyroid-specific diagnostic tests, incurred higher healthcare costs, and received more thyroid-related diagnoses, particularly non-toxic goiter. The data led researchers to determine that superfluous thyroid testing occurs frequently in everyday clinical settings, triggering a domino effect of additional interventions and costs. Their findings underscore the critical importance of developing more precise clinical guidance for thyroid US utilization. Such guidelines would serve multiple purposes: preventing patients from undergoing unwarranted invasive procedures, mitigating the psychological burden associated with unnecessary medical investigations, and addressing the significant financial impact on healthcare systems caused by excessive diagnostic testing [18].

Chen and colleagues described that US is frequently used for clinically unsupported reasons (i.e., patient request or abnormal results of thyroid function test), and showed that specialists are more likely to order US after patient requests [19]. In particular, as previously noted, thyroid nodules are frequently and incidentally discovered in patients who undergo unnecessary US exams. In turn, when additional (unnecessary) examinations are prompted, patients may face undue anxiety, and potentially overdiagnosis of thyroid cancer may occur. While the exact drivers behind inappropriate US use remain unclear, likely, a combination of clinician, patient, and healthcare system factors play a role [20]. Based on our experience, the main drivers of inappropriate thyroid US are education and examples given to young colleagues, missed continued education, differences in different guidelines, request of patients, economical reasons (revenue), US performed by the attending physician (self-referral) and “defensive” medicine. This review aims to explore patterns in US (mis)use in different clinical scenarios and provide potential strategies to mitigate overuse, which contributes to escalating healthcare costs and patient risks.

## 2. Thyroid Ultrasound: Fundamental Concepts

Thyroid US is an essential imaging tool for diagnosing thyroid disorders. This real-time, noninvasive method provides accurate evaluation of the thyroid gland and adjacent tissues, enabling detailed analysis of size, shape, composition, and blood flow. It is essential in identifying abnormalities, including nodules and diffuse thyroid diseases. The technology centers around an ultrasound probe (transducer) that emits high-frequency sound waves and detects returning tissue echoes, converting them into images. Transducer frequency selection (typically 5–15 MHz) varies based on patient body type and thyroid depth. Higher frequencies (10–15 MHz) deliver superior resolution for superficial structures, while lower frequencies (5–7.5 MHz) provide better penetration for deeper tissues. Linear array transducers are preferred for thyroid imaging due to their capacity to generate high-resolution images of the gland’s superficial regions [21]. Color Doppler US enhances conventional imaging by adding color-coded blood flow data within the thyroid. This technique helps identify hypervascular nodules, using red to show blood moving toward the transducer and blue for blood moving away, allowing assessment of flow direction and speed in vessels [22]. However, it has limitations in detecting artifacts and experiences challenges related to depth. Elastography is another complementary technique that measures tissue stiffness, thus helping distinguish between benign and malignant nodules. Tissue stiffness is quantified in kilopascals (kPa), with stiffer tissues often associated with malignancy. There are two main types of elastography: strain elastography, which offers a qualitative measure of stiffness based on tissue deformation, and shear wave elastography (SWE), which provides a more accurate, quantitative assessment. SWE is increasingly favored in daily practice due to its reproducibility and precision [23]

### Thyroid Ultrasound Examination Technique

The patient is placed in the supine position with an extended neck, facilitating optimal thyroid gland visualization. The examination proceeds by moving the transducer across the thyroid lobes and isthmus, evaluating both anterior and posterior aspects and lateral and medial margins. In addition to assessing nodules, the exam should evaluate surrounding structures such as lymph nodes and the parathyroid glands. Doppler technology can assess blood flow, while elastography may be applied to evaluate concerning thyroid nodules [24]. Ultrasound examination of the thyroid plays a crucial role in identifying, monitoring, and treating various thyroid conditions, such as nodules, inflammatory disorders, and other thyroid pathologies. It should be noted, however, that the diagnostic accuracy depends on the operator’s expertise, and errors in technique or interpretation may result in inaccurate diagnoses.

Thyroid US is currently performed by different specialists (i.e., radiologists, endocrinologists, nuclear medicine physicians, surgeons, and internal medicine specialists) with different technological and clinical competences. Generally speaking, physicians with a radiological background will pay greater attention to the technical (image formation, artifacts) and anatomical aspects, while those with a clinical background will find it easier to connect the clinical aspects to the US ones. Considering the increasing complexity of the instruments and the clinical scenario (for example, risk stratification of nodules), it is highly desirable to have a homogeneous definition (at least at a national level) of the requirements and the training path necessary for the execution and reporting of thyroid US examinations. As in every sector of medicine, experience and volume of activity influence the result: in particular, less experienced operators will be more inclined to emphasize even findings of little relevance (increasing false positives), while more experienced operators will be more confident in excluding relevant pathologies. Thyroid US remains, in any case, a highly operator-dependent method and, therefore, subject to significant inter-operator variability. While this is unlikely to have a significant impact in the case of functional diseases (see below), the diagnosis and typing of thyroid nodules may be negatively influenced. This problem was approached by creating the so-called Thyroid Imaging and Reporting Data System (TI-RADS) that contributed to a reduction in fine-needle aspiration procedures and ameliorated communications between different specialists. However, many different TI-RADSs are available, using different criteria and showing only a moderate inter-rater reliability (see below) [25]. Accordingly, a comprehensive approach that combines US findings with clinical evaluation remains crucial to ensuring the best patient outcomes.

## 3. Thyroid Ultrasound in Thyroid Dysfunctions

### 3.1. Hyperthyroidism

**Figure 1:**
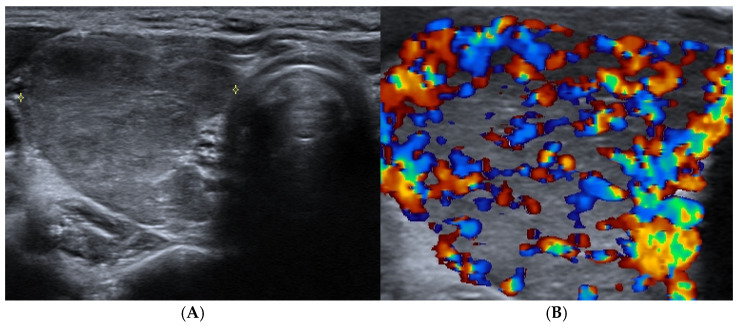
Graves’ disease: right thyroid lobe (axial view) with (**A**) reduced echogenicity and slightly heterogeneous texture and (**B**) diffuse and marked hypervascularity.

**Figure 2:**
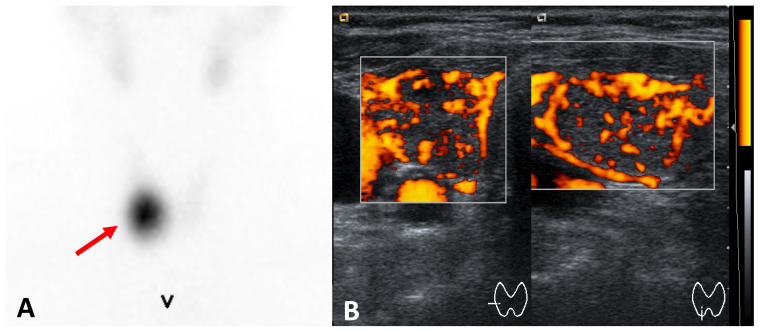
Toxic adenoma (right thyroid lobe) (**A**) ^99m^Tc-pertechnetate scintigraphy: hyperfunctioning nodule (arrow) and functional suppression of the remaining thyroid tissue; ultrasound ((**A**) axial, (**B**) longitudinal views): hypoechoic and well-demarkated nodule with increased vascularity.

**Figure 3:**
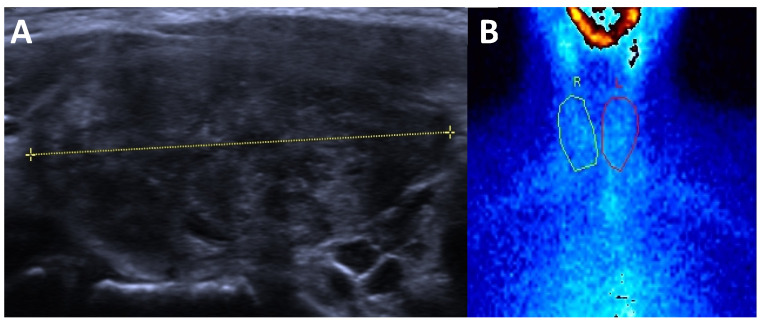
Subacute thyroiditis (**A**) ultrasound: enlarged thyroid that is markedly hypoechoic and hererogeneous and (**B**) ^99m^Tc-pertechnetate scintigraphy: absent thyroid activity.

Hyperthyroidism refers to an excessive level of thyroid hormones in tissues, resulting from increased hormone synthesis, excessive release of preformed thyroid hormones, or an exogenous or endogenous extrathyroidal source. The most prevalent causes of increased thyroid hormone production are Graves’ disease, followed by toxic multinodular goiter and toxic adenoma. In contrast, the most common cause of passive hormone release is painless (silent) thyroiditis, although its clinical presentation overlaps with other causes. Treatment of hyperthyroidism due to overproduction of thyroid hormones can include antithyroid medications (e.g., methimazole, propylthiouracil), radioiodine therapy, or thyroidectomy. Treatment selection depends on the underlying cause, potential contraindications, disease severity, and patient preferences [26]. Before initiating treatment, a comprehensive assessment is crucial to identify the precise etiology of hyperthyroidism. While a thorough clinical history and examination can often establish the cause, distinguishing Graves’ disease from other forms of thyrotoxicosis may pose challenges, especially in the absence of pathognomonic signs such as thyroid-associated orbitopathy or palpable thyroid nodules [27,28]. According to the American Thyroid Association guidelines, diagnosis can be confirmed with clinical evaluation and laboratory tests while imaging should be reserved to unclear cases. Radionuclide scanning, utilizing either ^99m^Tc-pertechnetate or ^123^I, remains the standard diagnostic approach for differentiating destructive thyrotoxicosis from hyperthyroidism and distinguishing between diffuse and focal thyroid hyperactivity [29,30,31]. Ultrasound patterns observed in patients with thyrotoxicosis can vary based on the underlying condition, such as Graves’ disease, toxic multinodular goiter, or thyroiditis but the test remains highly operator-dependent and is subject to considerable variability [32]. Radionuclide scan and US patterns of different causes of thyrotoxicosis are illustrated in Figure 1, Figure 2 and Figure 3.

Almost all available guidelines suggest the selective use of diagnostic tools to define the cause of thyrotoxicosis. However, significantly divergent recommendations can be found in the literature and clinical guidelines exist concerning the use of US that is recommended in all patients together with thyrotropin receptor antibodies (TRAb) by ETA guidelines, but only in patients with an unclear diagnosis and as an alternative to TRAb or scintigraphy by ATA. Finally, NICE guidelines suggest TRAb as the first step, followed by thyroid scintigraphy in TRAb-negative patients, while US is suggested only in the case of clinically detectable nodule(s) [33] (Table 1).

Measurement of TRAb is a highly effective and rapid diagnostic tool for Graves’ disease. Newer TRAb-binding immunoassays offer excellent sensitivity (>97%) and specificity (98–99%) [35]. Thyroid-stimulating immunoglobulins (TSI) serve as the hallmark of Graves’ hyperthyroidism and its extrathyroidal manifestations [36]. While previous methods of assessing TSI were complex and expensive, recent advances have simplified the process, enhancing their inclusion in diagnostic algorithms [37]. A study comparing diagnostic methods for thyrotoxicosis in 124 newly diagnosed, untreated patients (86 with Graves’ disease and 38 with non-Graves’ hyperthyroidism) found that thyroid scintigraphy remains the most accurate differentiation method. However, thyrotropin-receptor antibody assays (TRAb and TSI) could serve as an effective alternative, suggesting that thyroid scintigraphy might only be necessary for TRAb-negative patients. While thyroid US showed lower accuracy than both scintigraphy and antibody tests, the presence of a “thyroid inferno” pattern on US was found to have high positive predictive value for Graves’ disease [38]. All in all, it should be concluded that a careful clinical history and clinical examination are pivotal in evaluating patients with biochemically confirmed hyperthyroidism and may solve most cases without additional tests, reducing patients’ discomfort and anxiety as well as attached costs. Biochemical markers and imaging (US, thyroid scintigraphy, and radioiodine uptake test) should be selectively employed only in cases with an unclear clinical diagnosis. In unclear cases, a positive TRAb measurement confirms a GD diagnosis (the most frequent cause of hyperthyroidism) with exceedingly high accuracy. In TRAb-negative cases, thyroid scintigraphy offers the most accurate differential diagnosis (i.e., low uptake: destructive thyroiditis; uni- or multifocal hyperactivity: autonomously functioning nodules) and informs adequate treatments. Thyroid US cannot provide an accurate etiological/functional differentiation and should be limited to patients with clinically relevant non-autonomous nodules [34].

### 3.2. Hypothyroidism

In patients with suspected hypothyroidism, TSH measurement should be the initial diagnostic test. A normal TSH level generally rules out primary hypothyroidism. However, if clinical symptoms strongly suggest hypothyroidism despite non-elevated TSH, a free thyroxine level should be measured to evaluate for central hypothyroidism (caused by pituitary or hypothalamic dysfunction), which is much less common but should not be overlooked. Thyroid autoantibodies (antithyroid peroxidase, TPOAb, and antithyroglobulin antibodies, TgAb) are positive in 95% and 60% of patients with autoimmune thyroiditis, respectively. Testing for TPOAb provides adequate sensitivity and specificity to serve as the sole confirmatory test required for diagnosing autoimmune thyroiditis [39]. Importantly, up to 10% of the general population may be positive for TPOAb without having thyroid dysfunction, but treatment is not indicated in these individuals. On the other hand, it signals an increased risk of becoming hypothyroid over time, and annual TSH testing is suggested in this case [40]. In summary, thyroid imaging is not routinely indicated for a diagnosis of hypothyroidism alone. Thyroid US should only be performed when there are suspicious structural abnormalities, such as palpable thyroid nodules [41,42,43]. Despite consistent literature and guidelines suggesting a restricted use of US in patients with thyroid dysfunctions, current clinical practice diverges significantly from recommendations. Edwards and colleagues reviewed and meta-analyzed seven studies (total enrolled patients = 1573) and established an overall frequency of inappropriate US use of 46% (95% CI 15–82%; *n* = 388 patients) and 34% (95% CI 16–57%; *n* = 190 patients) in studies using guideline-based definitions. The pooled frequency of inappropriate US was 17% (95% CI 7–37%; *n* = 191 patients) in patients with thyroid dysfunction (either hypothyroidism or thyrotoxicosis) and 11% (95% CI 5–22%; *n* = 124) in patients with nonspecific symptoms without a palpable mass [16]. Acosta and colleagues reported that 10% to 50% of US orders are outside clinical practice recommendations. The drivers of inappropriate use of US are not yet completely defined, but a combination of clinician, patient, and healthcare system factors likely contribute to this problem [44]. Most data on the (in)appropriate use of US come from the United States, Canada, and the UK, while European data are not completely available. However, considering more permissive guidelines and the wide use of US in endocrinologists’ offices, the rate of inappropriately requested/performed procedures is likely higher in European countries. Notably, different clinical societies recently diffused a firm warning against the systematic use of US in patients with abnormal thyroid function tests (i.e., over- and under-active thyroid function). They stressed that it should not be required on a routine basis in patients with abnormal thyroid function tests but should be restricted to patients with large goiter or a lumpy thyroid at clinical examination. Specifically, they suggested that excessive reliance on US often reveals clinically insignificant nodules, potentially shifting the focus of medical evaluation away from the underlying thyroid dysfunction to these incidental findings. Finally, they suggest using thyroid scintigraphy to assess the etiology of the thyrotoxicosis when a differential diagnosis is needed, instead of US [45,46].

## 4. Thyroid Nodules

Thyroid nodules are distinct lesions within the thyroid gland that appear radiologically different from the surrounding thyroid tissue. They are a common finding, with a prevalence in the general population ranging widely from 2% to 65%, depending on which diagnostic techniques are used [47]. Furthermore, thyroid nodules occur with greater frequency in populations from iodine-deficient regions [48]. Despite the widespread implementation of iodized salt eliminating iodine deficiency in many global regions, the actual prevalence of thyroid nodules remains largely undetermined [49]. They are identified either through clinical examination (self-palpation by patients or examination by healthcare providers) or, much more commonly, as incidental findings during imaging procedures performed for unrelated reasons, such as US, computed tomography (CT), magnetic resonance imaging (MRI), or fluorodeoxyglucose positron emission tomography/computed tomography ([^18^F]FDG PET/CT). Indeed, US is still largely performed outside guidelines’ recommendations and without clear indications (as an example, see sections on thyroid dysfunctions), greatly increasing the number of detected nodules. Overall, the diagnosis of thyroid nodules includes a fraction that harbors malignancy (2–5%), may cause compressive symptoms (5%), or is functionally hyperactive (<5%) [50]. According to research by Chen and colleagues, thyroid cancer incidence significantly increased from 5.0 cases per 100,000 people in 1975 to 14.6 cases per 100,000 people in 2009, before stabilizing at approximately 14.1 cases per 100,000 people through 2019. This plateau was observed across nearly all age groups starting from age 25, with the most pronounced effect occurring in middle-aged adults (approximately 45–65 years), indicating a time-period rather than cohort effect. Consistent with findings from other regions, thyroid cancer mortality rates in the USA remained steady at 0.5 deaths per 100,000 people from 1975 to 2019. Importantly, the rate of metastatic disease at diagnosis also remained unchanged at 0.4 cases per 100,000 people during this same period [51,52].

### 4.1. How to Assess a Clinically Relevant Thyroid Nodule: The Role of Ultrasound

The first step in evaluating clinically relevant thyroid nodule is to measure the TSH level and perform US of the thyroid and cervical lymph nodes [53]. A normal or elevated TSH level indicates that a thyroid nodule is non-functioning, while a low or suppressed TSH suggests primary hyperthyroidism, warranting a radionuclide thyroid uptake scan. Hyperfunctioning nodules rarely harbor malignancy and do not require fine needle aspiration cytology (FNAC).

**Figure 4:**
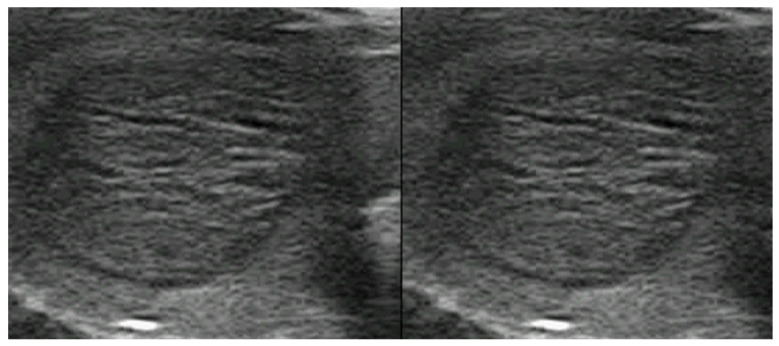
Typical benign spongiform nodule, where FNAC is not advised.

**Figure 5:**
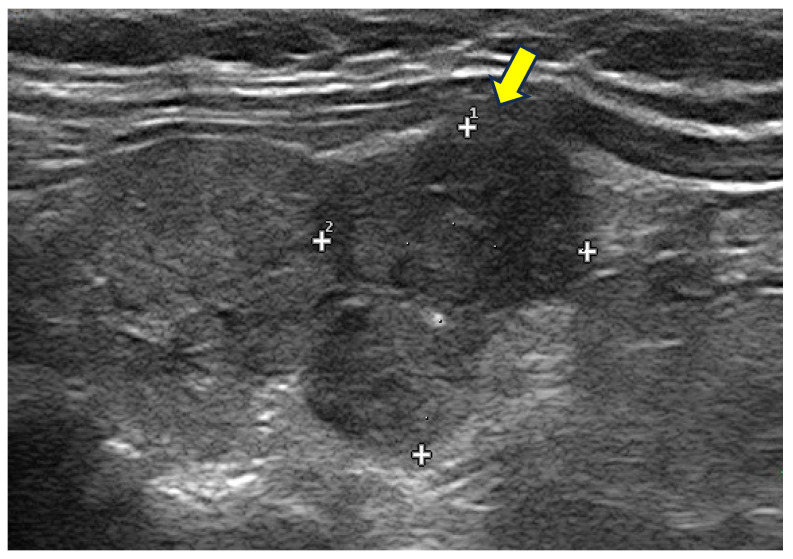
Hypoechoic, irregular nodules with focal capsule infiltration (arrow). Histology: papillary thyroid carcinoma of tall cell variant, with infiltration of surrounding soft tissue (pT3N0Mx).

Non-functioning or “cold” nodules should undergo FNAC if they meet specific clinical or US criteria. A thorough thyroid US report should document the gland’s parenchyma texture (hmogeneous/heterogeneous), overall size, and evaluate cervical lymph nodes in both central and lateral compartments for suspicious features. For each nodule, the report must specify three-dimensional measurements, exact location (such as right upper lobe), and detailed sonographic characteristics including composition (solid/cystic proportions or spongiform architecture), echogenicity, margin definition, calcification details, shape (particularly noting taller-than-wide orientation), and vascularity patterns. These comprehensive sonographic features, in conjunction with nodule size, are essential for malignancy risk stratification and guiding decisions regarding FNAC [54,55]. Due to the complex variations in US appearances across different thyroid cancer histologies and the diagnostic difficulties presented by partially cystic nodules, several researchers propose using a combination of integrated sonographic characteristics rather than isolated features for more effective risk stratification [56,57,58] (Figure 4 and Figure 5).

When suspicious cervical lymph nodes are not detected on US, high-risk sonographic features can help prioritize smaller thyroid nodules for FNAC. Conversely, nodules displaying lower-risk sonographic patterns may only warrant biopsy when they reach larger maximal diameters. Most thyroid nodules can be categorized into distinct US pattern classifications that incorporate multiple sonographic characteristics together, which helps address the moderate interobserver variability that exists when reporting individual features, even in controlled research settings [59,60,61]. Various Thyroid Imaging-Reporting and Data Systems (TI-RADSs) provide reliable frameworks for distinguishing between benign and malignant nodules, though they differ somewhat in their approaches. Among these systems, the American College of Radiologists TI-RADS (ACR TI-RADS) demonstrates superior diagnostic performance [62]. Higher scores correlate with increased malignancy likelihood, whereas lower scores suggest benign conditions. The combination of TI-RADS classification level and the nodule’s maximum diameter determines whether FNAC should be performed or regular monitoring is sufficient. Significantly suspicious nodules warrant biopsy only when they reach or exceed 1 cm in size. In contrast, low-risk nodules should undergo further investigation only upon reaching 2.5 cm or larger. Interestingly, research by Rucz and colleagues demonstrated that simply assessing echogenicity alone (recommending FNAC for all hypoechoic nodules regardless of other features) showed similar sensitivity and specificity to five different TIRADSs when identifying malignancies in nodules measuring between 10 and 20 mm [63]. A comprehensive examination of TI-RADS classification systems extends beyond the scope of our current paper. For more in-depth information, readers should consult recent reviews and comparative studies on this topic [64,65].

### 4.2. How to Manage a Thyroid Incidentaloma: An Open Debate

As detailed above, ITNs constitute a serious problem in terms of the induction of inappropriate diagnostic and therapeutic procedures, anxiety, patients’ discomfort, and costs [66]. Even if the best way to avoid ITNs is not to perform US without apparent clinical indications, the problem remains when such negligible nodules are detected during imaging procedures performed for other reasons. Song and colleagues performed an extensive literature review across PubMed, Embase, and Cochrane databases, identifying studies published before 12 April 2024, that reported prevalence, follow-up procedures, and risk of malignancy (ROM) in ITNs detected by CT. Their meta-analysis encompassed 38 studies with 195,959 patients for prevalence assessment, finding an overall ITN prevalence on CT of 8.3%. Prevalence was notably higher in neck CT (16.5%) than chest CT (6.6%). Multiple ITNs occurred in 27.0% of patients, with 46.3% of nodules measuring ≥1 cm and 28.6% measuring ≥1.5 cm. Follow-up procedures included thyroid US in 34.9% of cases, FNAC in 28.4%, and surgeries in 8.2%. Furthermore, data from 25 studies involving 6272 patients indicated a risk of malignancy of 3.9% for CT-detected ITNs. Notably, the ROM of nodules < 1 cm and <1.5 cm was negligible (0.1%, CI, 0–0.8 and 0%, CI, 0–0.2, respectively). This is perfectly in line with the recommendation not to perform FNAC in subcentimetric nodules, even if at high risk based on TI-RADS [67]. A considerable debate is ongoing, especially in radiology literature, with some data showing no excess risk in not reporting thyroid incidentalomas [68,69]. Considering the exceedingly high number of ITNs, the attached increase in incidental detection of small, smoldering, and clinically irrelevant thyroid carcinomas and flat mortality of thyroid cancer for decades, not reporting ITNs should be a safe and cost-effective solution [68,69]. However, it is likely that it will be difficult to accept for many reasons, including the absence of long-term prospective studies and potential ethical and medico-legal problems. As a consequence, ITNs are generally reported in clinical practice worldwide, generating a considerable volume of additional examinations with attached potential complications, costs, and patients’ discomfort and anxiety. In order to mitigate these problems and provide guidance to imaging specialists, the American College of Radiology formed the Incidental Thyroid Findings Committee to develop medically appropriate approaches to managing ITNs detected on different imaging modalities. CT or MRI-detected ITNs should first be evaluated for suspicious features, including abnormal lymph nodes or signs of local invasion, neither of which is likely in a patient without thyroid-related symptoms. For all patients exhibiting ITNs with suspicious characteristics, the Committee advises US verification of findings, followed by consideration of FNA. For patients with no suspicious imaging features of an ITN, comorbidities or limited life expectancy may increase the risk of morbidity and mortality more than the thyroid cancer itself. Hence, the Committee recommends that they do not undergo further evaluation. Exceptions include cases in which the referring clinician believes further evaluation is warranted, and in which the patient or referring physician specifically requests it. In the general population with normal life expectancy and without suspicious imaging features, patient age and nodule size should determine the need for workup. The Committee recommends further evaluation with the US for patients aged <35 years with nodules measuring 10 mm or more in the axial plane. If the patient is aged 35 years, the size cutoff in the axial plane for further evaluation is raised to 15 mm. In cases of multiple thyroid nodules, this flowchart should be applied to the largest (i.e., dominant) nodule [70].

### 4.3. PET/CT Incidentalomas: A Particular Case

As 18F-fluorodeoxyglucose positron emission tomography [^18^F]FDG PET/CT becomes more commonly used for cancer investigation and staging, thyroid nodules with [^18^F]FDG uptake are increasingly discovered incidentally, occurring in approximately 1–4% of [^18^F]FDG PET/CT scans. These incidental [^18^F]FDG-avid thyroid nodules carry a malignancy risk of 15–20%, substantially higher than nodules found through US, CT, or other imaging methods. Nevertheless, when malignancy is confirmed, most cases represent differentiated thyroid cancers with excellent outcomes even without intervention. Therefore, management decisions should carefully consider the patient’s primary cancer diagnosis, age, and existing health conditions, as further investigation of an incidental [^18^F]FDG-avid thyroid nodule may often be unnecessary. Wadsley et al. developed a consensus statement derived from both a literature review and the clinical expertise of the multidisciplinary panel. They recommended to firstly consider the patient’s age, long-term prognosis, comorbidities, and PET characteristics of the thyroid nodule, taking into account the patient’s understanding of their prognosis and any uncertainty around this, when deciding whether to investigate a thyroid incidentaloma. If it is likely that the patient will not survive 5 years, further investigation is unlikely to be appropriate. Risk stratification with US is deemed to be appropriate in patients with an [^18^F]FDG-avid TNI who have a reasonable life expectancy (greater than 5 years). In such cases, the decision to perform or not to perform an FNAC should be based on US appearances. In summary, FNAC is not indicated in nodules of any type with a diameter less than 10 mm or in nodules with an unsuspicious US pattern (i.e., EUTIRADS 2). An FNAC should be considered in [^18^F]FDG-active nodules > 10 mm with US intermediate risk (i.e., EUTIRADS 3), while it is recommended in nodules with higher risk [71].

The AJR Committee’s guidance for managing ITNs detected on [^18^F]FDG PET scans suggests thyroid US and FNAC, if needed, in the general population of patients. On the other hand, no further evaluation of the ITN is recommended in patients with severe comorbidities or limited life expectancy [70].

### 4.4. Cost-Effectiveness and Suggested Recommendations

The excessive ordering of unnecessary or inappropriate medical tests constitutes overuse and represents inefficient resource allocation. In 2012, Berwick and Hackbarth estimated that 6% to 8% of U.S. annual healthcare expenditures (at least USD 270 billion) could be categorized as overuse [72]. While the global burden of thyroid nodules has been frequently reported, the associated economic burden is partially understood. For malignant nodules, the costs of well-differentiated thyroid cancer care in the United States are projected to exceed USD 3.5 billion by 2030 [73].

**Figure 6:**
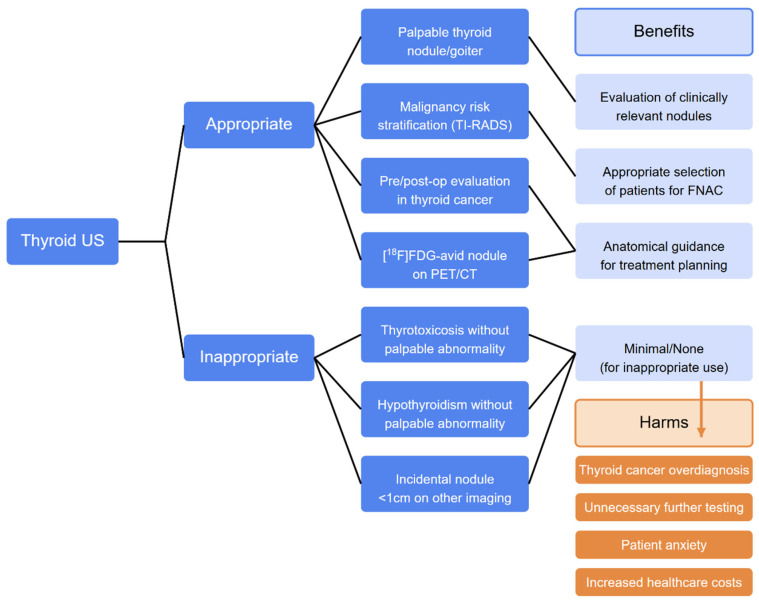
Flowchart in approaches to thyroid US. Legend: FNAC, fine needle aspiration cytology; TI-RADSs, Thyroid Imaging Reporting and Data Systems; [^18^F]FDG, fluorodeoxyglucose; PET/CT, positron emission tomography/computed tomography.

Prior cost-effectiveness analyses estimated that the screening and management of all thyroid nodules in the United States would incur USD 25.1 billion in costs, and the addition of specific biomarkers, such as serum calcitonin for medullary thyroid cancer, to current ATA guidelines could represent an increase of USD 1.4 billion in costs, which would result in a mean value of USD 11,793 per life-year saved [74]. For ITNs smaller than 2 cm, FNAC demonstrates poor cost-effectiveness when compared to observation (USD 542 vs. USD 412 in direct costs) [75]. Studies examining medical test overutilization in both hospital and ambulatory settings have prompted initiatives such as Choosing Wisely, the Right Care Alliance, and Wiser Healthcare [76]. The panel emphasized that performing US indiscriminately identifies numerous thyroid nodules without clinical significance, potentially causing patient anxiety, increasing diagnostic and surgical interventions (possible overtreatment), leading to unjustified community costs, and risking patient harm. Therefore, recommendations include the following: (1) avoiding US screening in populations not at risk for thyroid cancer; (2) avoiding frequent US monitoring in subjects with chronic autoimmune thyroiditis without nodules; and (3) limiting FNAC to nodules classified as higher-risk based on US risk stratification. Additionally, thyroid US should not be routinely ordered for patients with abnormal thyroid function tests unless there is a palpable thyroid abnormality. Despite such efforts, the situation is far from optimal, and based on our experience, almost all patients with thyroid dysfunctions receive one or more US examinations, and it is relatively rare to receive patients with a nodule discovered by a clinical examination. Several factors contribute to this problem, including misrepresentation of weak evidence, leading many healthcare providers and patients to incorrectly believe that more testing invariably leads to better outcomes by potentially identifying treatable conditions. In clinical settings, physicians frequently order routine tests for various reasons, including self-referral practices [77]. Current indications for US in patients with thyroid dysfunctions are summarized in Table 2, and a proposed flow chart is illustrated in Figure 6.

Ultrasound-induced thyroid cancer overdiagnosis leads to unnecessary treatments, putting patients unnecessarily at risk for the potential morbidity and mortality that may arise from those treatments, and it wastes healthcare resources. Patients may also suffer unnecessary anxiety, job discrimination, financial hardships, and other detrimental effects on quality of life. Li and colleagues showed how psychological distress and sleep disturbance increase after detection of a thyroid nodule, even if most nodules are clinically insignificant. All in all, overdiagnosis of mostly benign, indolent nodules results in negative effects on the patient’s psychological health [78]. Accordingly, Kornelius and colleagues identified a significant association between thyroid nodules and increased anxiety risk. Compared to patients with thyroid cancer, the risk of depression, mood disorders, and insomnia was lower. Furthermore, a sensitivity analysis compared patients with benign thyroid nodules to the general population and revealed elevated anxiety risk in patients with nodules, reinforcing that this increased risk is not solely attributable to cancer-related factors [79].

All in all, available data underscore the independent psychological burden posed by benign nodule surveillance. Accordingly, clinicians should provide comprehensive education, adequate information on the pros and cons of performing thyroid US, and reassurances regarding the typically benign nature of most nodules and the indolent behavior of most thyroid cancers.

## 5. Conclusions

While thyroid US is integral in evaluating patients with clinically relevant nodules, assisting FNAC procedures, assessing neck lymph nodes and monitoring thyroid cancers, the current overdiagnosis of thyroid nodules and clinically irrelevant cancer stems primarily from the improper use of US, which increases healthcare expenses and potential patient harm (inappropriate interventional procedures and surgeries with attached side effects and complications, psychological distress and anxiety). This inappropriate use results from inadequate knowledge and implementation of guidelines, widespread availability of US equipment in various specialists’ offices (with associated self-referral), the quick and uncomplicated nature of the procedure, and growing patient demand. Addressing this overutilization requires a better understanding of how frequently US is inappropriately used clinically and identification of the contributing factors. Such data would enable the development of targeted interventions to reduce unnecessary US examinations, ultimately enhancing patient outcomes and optimizing healthcare resource allocation. For now, we strongly suggest refraining from performing US in hyperthyroid and hypothyroid patients without palpable nodules and euthyroid patients without palpable abnormalities. No additional studies should be required in patients with subcentimetric nodules incidentally detected during non-thyroid-directed imaging procedures. With this simple and well-supported action, the number of US cases will decrease without harm to our patients, leaving the rare patients with relevant thyroid cancers to have the best service in terms of diagnosis and treatment. Therefore, it probably is not the time to shut down our US machines, but it is time to thoroughly the best course of action before moving the probe.

## Figures and Tables

**Table 1:** Current recommendations on hyperthyroidism diagnostics.

Reference	Scintigraphy	TRAb	Ultrasound
ETA 2018 [28]	II line *	I line	I line
ATA 2016 [27]	II line **	II line **	II line **
NICE 2023 [34]	II line ***	I line	II line *

Legend: ETA, European Thyroid Association; ATA, American Thyroid Association; NICE, National Institute for Health and Care Excellence; * clinically detected nodule(s); ** diagnosis not clinically evident (alternative use of different tools); *** if TRAb negative; TRAb, thyrotropin-receptor antibodies.

**Table 2:** Indications to perform a thyroid ultrasound in patients with thyroid dysfunctions.

Clinical Scenario	Indication	Recommendation
Thyrotoxicosis/Hyperthyroidism		
Unremarkable clinical examination	Diagnostic evaluation	Not recommended
Palpable goiter/nodule(s)	Anatomical evaluation	Recommended
TRAb positive	Differential diagnosis	Not recommended (TRAb confirms Graves’ disease)
TRAb negative	Differential diagnosis	Consider thyroid scintigraphy instead
Hypothyroidism		
Unremarkable clinical examination	Diagnostic evaluation	Not recommended
Palpable goiter/nodule(s)	Anatomical evaluation	Recommended
TPOAb positive	Etiological diagnosis	Not recommended (TPOAb confirms autoimmune thyroiditis)
Euthyroid state		
Unremarkable clinical examination	Screening	Not recommended
Palpable goiter/nodule(s)	Anatomical evaluation	Recommended
Incidental nodule < 1 cm on other imaging platforms	Follow-up	Not recommended
Incidental nodule ≥ 1 cm on other imaging platforms	Risk stratification	Consider [TI-RADS]
[^18^F]FDG-avid thyroid nodule on PET/CT	Risk assessment	Consider with clinical context (higher malignancy risk)

Legend: TRAb, thyrotropin-receptor antibodies; [^18^F]FDG, fluorodeoxyglucose; PET/CT, positron emission tomography/computed tomography; TI-RADSs, Thyroid Imaging Reporting and Data Systems.
